# Umbilical Cord Segmental Hemorrhage and Fetal Distress

**Published:** 2006-06

**Authors:** Giovanni Larciprete, Maria Elisabetta Romanini, Domenico Arduini, Elio Cirese, Jolanta Slowikowska-Hilczer, Krzysztof Kula

**Affiliations:** 1 *A. Fa. R. Associazione Fatebenefratelli per la Ricerca, Isola Tiberina, Roma, Italy;*; 2 *Perinatal Medicine, Tor Vergata University, Rome, Italy*

**Keywords:** umbilical cord, amniotic fluid, fetal distress

## Abstract

We describe an unexplained case of umbilical cord segmental hemorrhage linked with meconium-stained amniotic fluid. A severely asphyxiated infant was delivered at term by Caesarean section. There were poor prognostic signs on fetal cardiotocography with rupture of membranes with meconium-stained amniotic fluid. The pathophysiologic mechanism in this case is still unknown, even if we argued a possible role of the umbilical cord shortness.

## CASE

**Figure 1:**
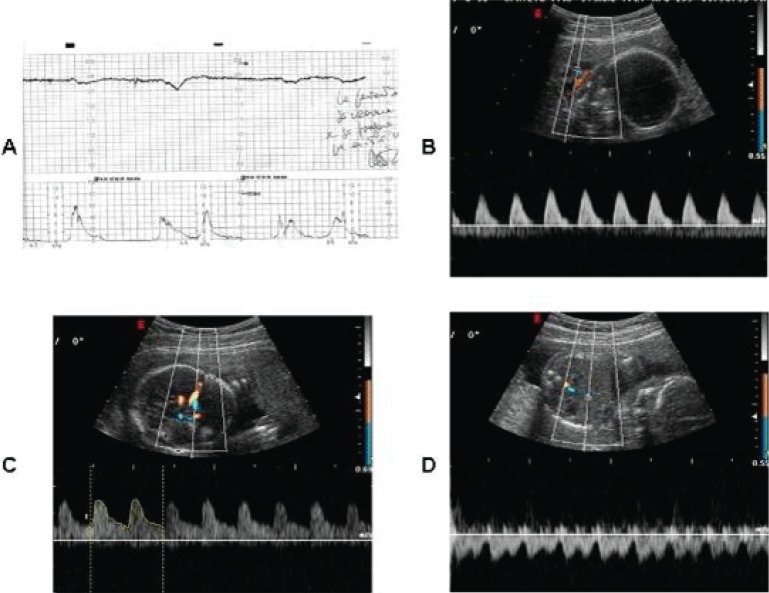
A. Fetal heart rate monitoring: decreased variability and tachycardia. B. Pulsed Doppler evaluation of the umbilical artery showing absence of the end-diastolic flow. C. Pulsed Doppler waveform of the ductus venosus at 39 weeks gestation showing reversed end-diastolic blood flow (during atrial contraction). D. Doppler sonography of the middle cerebral artery showed reduced resistance.

On February 2, 1999, a 31-year-old woman, gravida 1 at 39 weeks’ gestation, was admitted to Fatebenefratelli Hospital (Tor Vergata University, Rome) with fetal tachycardia (170 beats/minute, Figure 1), little variability in fetal heart rate from cardiotocography (amplitude range of 5 beats/minute), increased decelerations (lasting 5 minutes) and rupture of membranes. Thereafter, an ultrasound scan was performed, which showed an absence of end-diastolic flow of the umbilical artery (UA), abnormal ductus venosus (DV) flow and reduced cerebral vascular resistances (Figure 1). From the clinical history, we found that the patient had had a 6-hour history of strongly brown stained and highly viscous amniotic fluid loss.

Within 25 minutes of admission, a Caesarean section was performed for acute fetal distress, since a spontaneous vaginal delivery was not imminent. A severely asphyxiated male newborn was delivered, weighing 3020 grams. He had low Apgar scores (4 and 5 at 1 minute and 5 minutes respectively) with an umbilical arterial pH of 6.9. The neonate did not breath spontaneously and was promptly intubated and ventilated and transferred to the neonatal intensive care unit where 5 days of supportive management was required.

**Figure 2:**
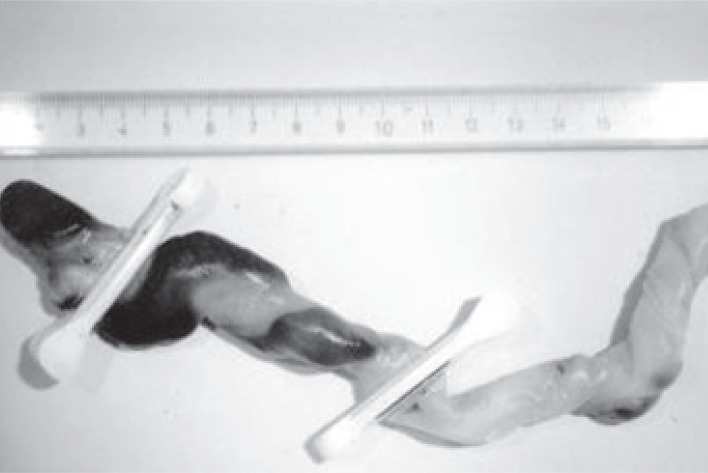
Umbilical cord specimen: 10 cm black-colored tract following the vessels with a spiral shape.

**Figure 3:**
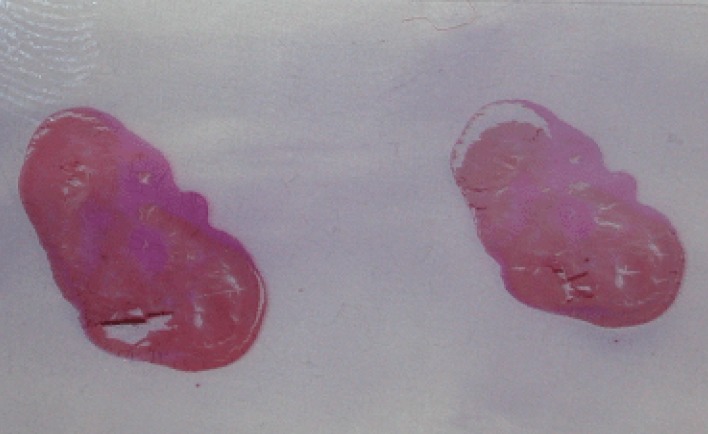
Gross examination of the slide with the fixed and colored specimen, showing hemorrhage surrounding and pressing the umbilical cord vessels.

**Figure 4:**
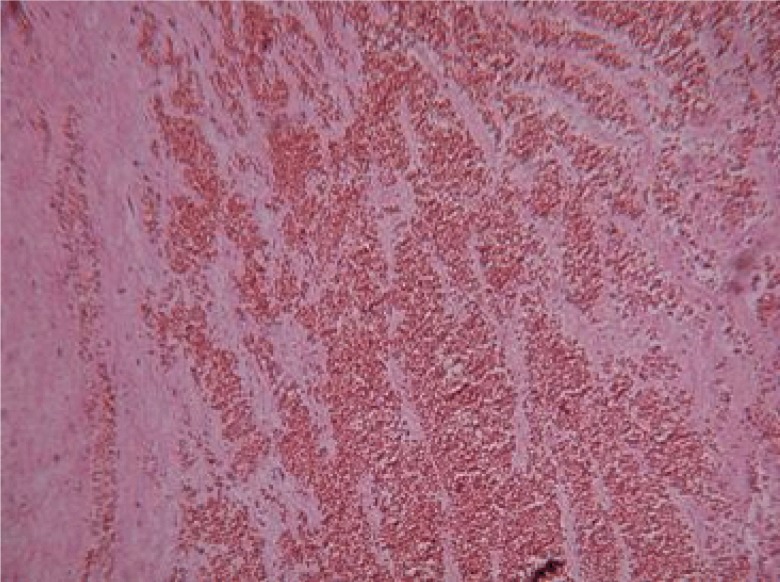
Light microscopy. Fetal red blood cells into the stroma. Vessels walls are squeezed by the outstanding infarction.

The placenta weighed 650 g and cord measured 34 cm. There was uniform meconium staining of the umbilical cord and the fetal membranes. Serial cross-sections of the blood vessels failed to disclose any evidence of thrombi, kinking or torsion. The umbilical cord was in part black-colored in a tract measuring 5 cm (Figure 2). Gross examination of the fixed specimen clearly demonstrated an infarction of the cord (Figure 3). On light microscopy, an umbilical cord segmental hemorrhage was noted, with prominent fetal red blood cells seen around the vessels (Figure 4), fitting the Wharton’s jelly in the perivascular space, by a 10 cm length (Placental Pathology). All microbiological cultures of the placenta and membranes were negative.

Both mother and neonate were discharged from hospital after 10 days without further complications. The neonate was followed up and remains in good health after 10 months of delivery.

## COMMENT

Bleeding from the vessels of the cord is usually the result of obstetric trauma either at the time of delivery or occasionally as a result of puncture during diagnostic amniocentesis (2).

The vessels at the velamentous insertion are at particular risk during delivery as they lay unprotected. Additonally, a very short umbilical cord may be torn or avulsed during the second stage of labor. In general, haemorrhage into the substance of the cord is usually venous in origin, although if extensive it may surround all three vessels and result in compression, with the most impact seen on venous flow.

The incidence of true antepartum hematomas is low: 1 in 5505 according to Dippel (3). In general, it is difficult to be certain that the cord hemorrhages found on routine examination of the placenta are significant, since the majority will be the result of handling during labor. The lesions are usually segmental, measuring up to 10 cm in length though up to 42 cm of cord have been described. Beside trauma, other etiological factors include inflammation, abnormal vessel structure, varicosities, syphilis and idiopathic calcification may be involved.

Previously, Feldberg *et al*. (4) described a spontaneous haematoma of the umbilical cord where no pathologic lesion was found within the vessels. However, they speculated that an extremely short cord (14 cm) may have contributed to the vessel rupture. A prolonged deceleration discovered during a routine nonstress test led to emergency Caesarean section, with delivery of a healthy neonate.

In our case the cord was short too, leading us to follow this aetiologic factor, like described elsewhere (5, 6)

Gregora and Lai (7) showed that fetal heart rate monitoring and ultrasound is critical in the management of such a rare disease, following the observations made by Feldberg. From our case, we hypothesize that the occurrence of a late deceleration during non- stress cardiotocography is the epiphenomenon of an extremely rare event, rather than a practical and effective diagnostic tool to detect such a detrimental lesion. However, it is important to mention that previous authors have also highlighted the importance of poor prognostic signs on fetal heart rate when managing fetuses with cord damage (5, 6).

The mortality associated with this umbilical lesion is high and up to fifty percent usually die (8, 9). From this, it is clear that umbilical cord puncture should always be undertaken with extreme care when unexplained intrauterine death has occurred following amniocentesis. In our case, it appeared that none of the previously described etiological factors were involved. Additionally, no trauma occurred during labor.

Only the mild shortness of the umbilical cord could be taken into account as a possible determining factor for such a rare case.
